# Effects of Conservation Tillage on Soil Physicochemical Properties and Crop Yield in an Arid Loess Plateau, China

**DOI:** 10.1038/s41598-020-61650-7

**Published:** 2020-03-13

**Authors:** Juan Li, Yi-ke Wang, Zhen Guo, Jin-bin Li, Chang Tian, Dong-wen Hua, Chen-di Shi, Huan-yuan Wang, Ji-chang Han, Yan Xu

**Affiliations:** 1 Institute of Land Engineering and Technology, Shaanxi Provincial Land Engineering Construction Group Co., Ltd, 710075 Xi’an, China; 2 Shaanxi Provincial Land Engineering Construction Group Co., Ltd, 710075 Xi’an, China; 3 Key Laboratory of Degraded and Unused Land Consolidation Engineering, the Ministry of Land and Resources, 710075 Xi’an, China; 4 Shaanxi Provincial Land Consolidation Engineering Technology Research Center, 710075 Xi’an, China

**Keywords:** Geochemistry, Environmental impact

## Abstract

Conservation tillage can improve soil physical structure and water storage, protect moisture, and increase crop yield. However, the long-term adoption of a single tillage method may have some adverse effects on soil and ecological environment, although crop yields have increased. Through informed allocation of soil tillage techniques, the combination and configuration of soil tillage measures, such as rotary tillage, subsoiling, and no tillage may reduce the shortcomings of traditional long-term farming. To explore the long-term production mode suitable for production of maize in the loess dryland area, a long-term experiment was conducted in Fuping County, Shaanxi Province, from 2013 to 2018. Six farming modes were used in the experiment: no tillage/subsoiling (N ↔ S), subsoiling/rotary tillage (S ↔ R), rotary tillage/no tillage (R ↔ N), continuous no tillage (N ↔ N), continuous subsoiling (S ↔ S), and continuous rotary tillage (R ↔ R). The changes in soil physical and chemical properties, soil water use patterns, soil water storage, conservation effects during the fallow and growth period, and the effects on farmland yield increase were analyzed. The results showed that rotary tillage can effectively improved soil structure and reduced soil bulk density, where N ↔ S treatment soil bulk density is low and in 0–60 cm soil layer averaged 1.31 g/cm^3^. Different tillage treatments could be used during the fallow period to store additional soil moisture: the N ↔ S treatment showed good water storage effect. Compared to traditional tillage, different tillage methods provided better soil moisture conditions for crops during the growth period, where N ↔ S treatment showed good soil moisture status during the growth period of spring maize. Among all the treatments, N ↔ S treatment effectively increased the organic carbon storage in the 0–60 cm soil layer, which was 54.3 t/hm^2^. Compared with traditional tillage, different tillage treatments effectively increased plant height and dry matter accumulation of spring maize, where N ↔ S treatment was found to be the best. Compared with the traditional rotary tillage model, the N ↔ S treatment significantly increased crop yield and water use efficiency (WUE) in continuous cropping fields of corn, the average yield of spring corn was 9340.2 kg/hm^2^, and the average WUE was 22.9 kg/(hm^2^·mm). In summary, for long-term sustainable development, the N ↔ S model is the best rotational tillage mode for continuous maize cropping in loess soil.

## Introduction

The loess tableland area is located in the south-central part of the Loess Plateau. It is a transitional area between the gully region of the Loess Plateau and the valley plain, and spans Shaanxi and Shanxi Provinces^1^. Grain production is dominated by winter wheat and spring maize; the shortage of nutrient and water restricts high and stable grain yields^2^. Conservation tillage uses the combined effects of mechanical force, biological force and wind power to achieve the exchange and balance of water, fertilizer, gas, and heat. Measures such as loosening, no tillage, and crop straw can significantly reduce wind and water erosion, increase soil organic carbon (SOC) content, improve soil water storage capacity, and water use efficiency (WUE) and protect ecological environments. The development of sustainable agriculture is of great significance. According to Piece *et al*.^3,4^, regular soil tillage after no tillage can improve soil physical properties, reduce soil bulk density, and increase soil total porosity. Carter *et al*.^5^ showed that, compared with continuous no tillage and continuous subsoiling, no tillage and subsoiling combined rotation tillage measures were more effective in maintaining soil physical properties. López-Fando *et al*.^6^ also found that compared with continuous no tillage, rotary tillage can improve the physical and chemical properties of the soil. Vetsch *et al*.^7^ showed that, compared with long-term no tillage and strip tillage, subsoiling tillage and strip tillage maximized maize and soybean yields. The conservation tillage technique based on straw-covered no tillage and subsoiling in the fallow period can effectively reduce soil disturbance of the plow layer, increase the surface cover and soil organic matter content^8,9^, and promote the storage of soil moisture. To increase the yield and quality of crops, and the water storage and fertility of soil in the loess dryland area, it is necessary to reduce the threat of wind erosion and improve the quality of cultivated land^10,11^. Liu *et al*.^12^ stated that conservation tillage can increase maize and wheat yield by 11.8% and 9.7%, respectively. However, with the continuous research and application of conservation tillage, researchers have found that no-tillage can only adapt to some soils and natural conditions^13^. After years of no tillage, the soil becomes hard and bulk density increases, which affects the development of crop roots and the absorption of water and nutrients^13^. In recent years, researchers have suggested that timely implementation of soil rotation measures, such as subsoiling, no tillage, and tillage, and the establishment of soil rotation technology systems are effective measures to solve the shortcomings of long-term continuous single farming measures^14,15^. At present, the research on the effect of rotation tillage technology mainly focuses on the double-season rice areas in south China and the two mature areas in north China. Forming a soil rotation model that matches the planting system in the planting area is an effective way to improve soil quality and solve low crop yields. However, the research cycle of rotation cultivation technology is relatively long and long-term positioning tests are difficult, and there is less research on this aspect at this stage^16^. Our 5-year soil rotation tillage experiment was carried out in fall 2013 and was conducted in a continuous cropping maize field in the Weibei dryland area. To explore the effects of six rotation tillage modes on soil bulk density, soil moisture, organic carbon storage, plant height, biomass, grain yield and water utilization in experimental fields after the maize stalks are all returned to the field. The purpose was to evaluate and screen the optimal protective rotation mode with good structure, sufficient fertility and high yield, and provide scientific support and theoretical basis for the establishment of the rotation tillage model matching with the local crop planting system. The conservation tillage model with good effect of water storage, soil conservation and yield increase can optimize the soil structure, drive farmers’ income, and promote regional economic development.

## Results

### Soil bulk density

**Figure 1:**
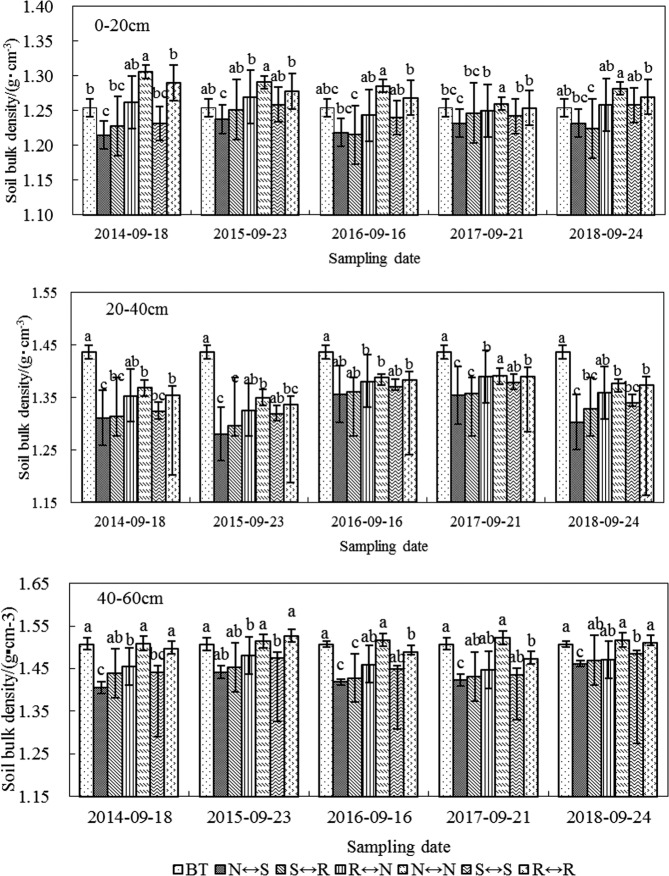
Effect of different rotational tillage treatments on soil bulk densit at 0–60 cm soil layer of spring corn field after harvesting.

Soil bulk density is the main physical indicator reflecting soil structure, gas permeability, water permeability, and water retention capacity. With the increase in soil depth, soil bulk density of each rotation mode showed an increasing trend. That is, the average soil bulk density of the 6 tillage patterns in the 0–20, 20–40, and 40–60 cm soil layers was 1.3, 1.4, and 1.5 g/cm^3^, respectively, and there was a significant difference in soil bulk density between different tillage models (P < 0.05), as shown in Fig. 1.

Because of the different soil cultivation methods used in the spring maize planting year under the RT mode, the soil bulk density of the 0–20, 20–40, and 40–60 cm soil layers showed an interannual variation trend. The soil bulk density of each soil layer in N ↔ S, S ↔ R and S ↔ S treatments decreased to varying degrees compared with BT treatment, the average decrease of bulk density of 0–20 cm soil layer was 2.16%, 1.67% and 0.63%, respectively (P < 0.05). Compared with BT treatment, the soil bulk density of R ↔ N, N ↔ N and R ↔ R treatments under the 0–20 cm soil layer showed an increasing trend, and the increase rates were 0.2%, 2.5%, and 1.4%, respectively (P < 0.05).

In the five experimental years, compared with before the experiment, the soil bulk density with N ↔ S, S ↔ R, R ↔ N, N ↔ N, S ↔ S, and R ↔ R tillage treatments in the 20–40 cm soil layer was decreased by 8.1%, 7.3%, 5.2%, 4.3%, 6.3% and 4.8%, respectively. Soil bulk density decrease was most significant in N ↔ S (P < 0.05). In the 40–60 cm soil layer, the soil bulk density of N ↔ S, S ↔ R, R ↔ N, and S ↔ S decreased with time, which were 5.2%, 4.2%, 2.9%, and 3.3%, respectively. N ↔ N increased by 0.6% compared with the soil bulk density before the test.

### Soil water storage during fallow periods

**Figure 2:**
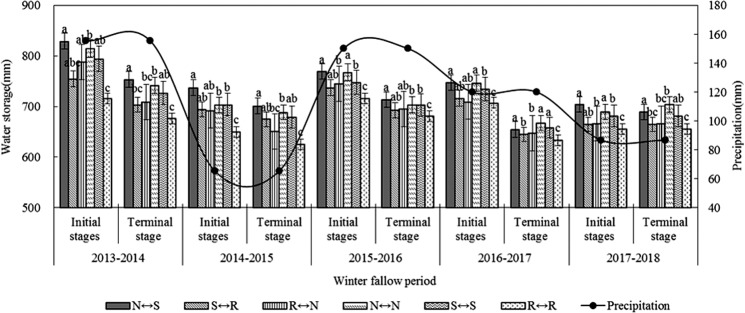
Effect of different rotational tillage treatments on soil water storage at 0~300 cm soil layer of during leisure periods.

Different tillage treatments during the rest period could effectively increase the soil water storage capacity of farmland (Fig. 2). The average soil water storage in the 0–300 cm soil layer at the end of different tillage activities was N ↔ S (705.4 mm)> N ↔ N (698.1 mm)> S ↔ S (689.4 mm)> S ↔ R (676.2 mm)> R ↔ N (67.3.6 mm)> R ↔ R (654.3 mm). The average soil water storage in each 0–300 cm soil layer was higher than that in the traditional tillage R ↔ R treatment. At the end of the 2013–2014 trial year, the soil water storage capacity of N ↔ S, S ↔ R, R ↔ N, N ↔ N, and S ↔ S was significantly higher than that of the control R ↔ R treatment, and increased by 11.5%, 4.1%, 4.9%, 9.7%, and 7.5%, respectively. In 2014–2015, because of lower rainfall during the rest period, the soil water storage capacity of each treatment was lower than that of the previous rest year, which was 4.0% to 12.1% higher than that of the control R ↔ R treatment. Compared with R ↔ R, the soil water storage capacity of N ↔ S, S ↔ R, R ↔ N, N ↔ N and S ↔ S increased by 4.8%, 1.8%, 2.1%, 3.4% and 3.2% respectively in 2015–2016, the soil water storage capacity of N ↔ S, S ↔ R, R ↔ N, N ↔ N and S ↔ S was increased by 3.4%, 1.8%, 2.2%, 5.3% and 3.9%respectively in 2016–2017, the soil water storage capacity of N ↔ S, S ↔ R, R ↔ N, N ↔ N, and S ↔ S was increased by 7.3%, 1.4%, 1.6%, 5.1% and 3.9% respectively in 2017–2018. In different rest years, the water storage effect in the N ↔ S rotation mode was better.

### Soil water content during fallow periods

**Figure 3:**
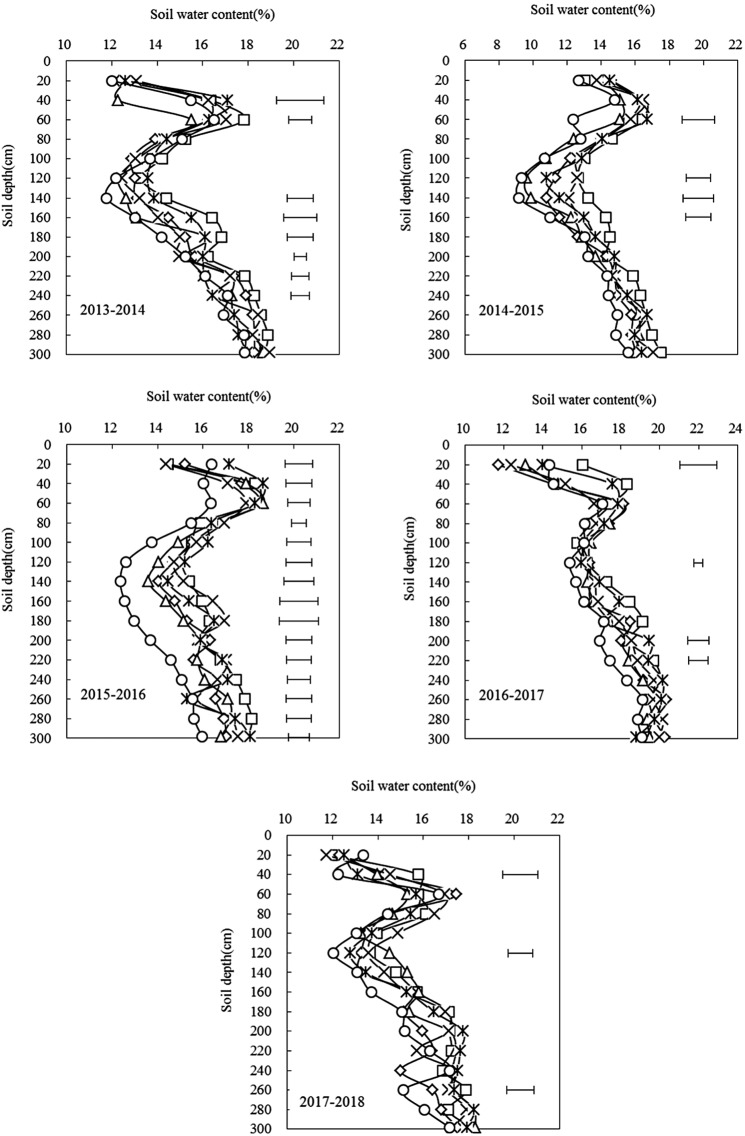
Effects of different tillage treatments on soil water content in 0–300 cm depth during the leisure period of maize field.

Figure 3 shows the dynamic change in water content in the soil profile of 0–300 cm soil layer during different interannual fallow periods. The soil water content during the rest period was greatly affected by precipitation, and the difference varied among years. The soil water content of each treatment in the 0–300 cm profile soil has a similar trend, with a small increase in the 0–60 cm soil, a decrease in the 60–120 cm soil, and an increase in the 120–300 cm soil, but the soil water content in the 40–60 cm soil layer of each treatment has always maintained a high level.

In 2013–2014, the rainfall during the rest period was relatively high, and the soil water content was as well. The soil water content ranged from 12.0% to 18.9%; the difference between treatments was significant (P < 0.05). In the 0–300 cm soil layer, the average water content for each treatment soil was N ↔ S > S ↔ S > S ↔ R > N ↔ N > R ↔ N > R ↔ R, and the average water content of other treated soils was higher than that of the control R ↔ R. The R treatment increased from 0.6% to 9.4%. In 2014–2015, the rainfall during the rest period was relatively low, and the soil water content was relatively low. The soil moisture content fluctuated from 9.15% to 17.48%. The soil moisture content was the highest in the N ↔ S treatment, followed by N ↔ N. In the 40–60 cm soil layer, the difference between treatments was significant (P < 0.05). The soil moisture content of N ↔ S, S ↔ S, and N ↔ N treatments was significantly higher than that of the control R ↔ R. In 2015–2016, the rainfall during the rest period was relatively high, and the soil water content was relatively high. The soil moisture content fluctuated from 12.4% to 18.7%. In the 60–300 cm soil layer, the soil moisture content under each tillage treatment was significantly higher than the R ↔ R treatment, and the differences between the treatments reached a significant level. In 2016–2017, the rainfall during the rest period was relatively high, and the soil water content was relatively high. The soil moisture content fluctuated from 11.7% to 19.7%. The significant difference in soil water content between tillage treatments only occurred at 0–20 cm. At the 20–40 cm and 200–260 cm soil layers, the average water content of N ↔ S treated soil was the highest, and the difference was significant compared with that of the control R ↔ R. In 2017–2018, the soil moisture content fluctuated from 11.7% to 17.5%; the soil moisture content in the treatment was significantly different between 20–40 cm, 100–120 cm, and 240–260 cm. During the whole experiment, the water content of the N ↔ S treatment was higher than that of the other tillage treatments.

### Soil water storage during growing periods

**Figure 4:**
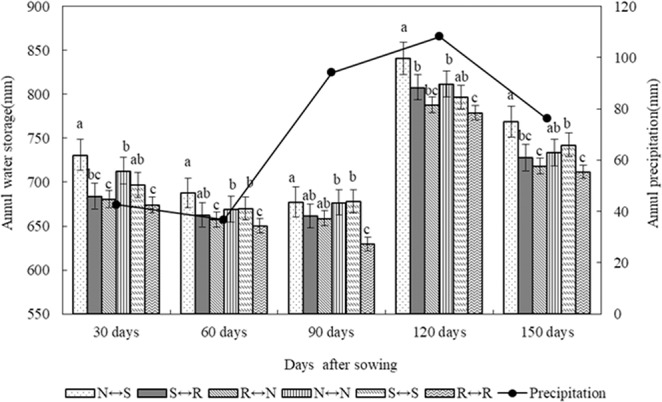
Soil water storage changes in 0–300 cm soil layer of different tillage treatments after the sowing of maize field in 2014–2018.

Dynamic changes in water during crop growth significantly affects crop yields. Through analysis of five test years, during the growth period of spring maize, the soil water storage in the 0–300 cm soil layer first decreased, then increased, and then decreased at the harvesting period (150 days after sowing) (Fig. 4), mainly because of the concentration of rainfall. During the entire spring maize growth period, soil water storage was higher N ↔ S, S ↔ S, and N ↔ N, and there were significant differences between the treatments and the control R ↔ R treatment (P < 0.05). During the entire growth period of spring maize, the average water storage capacity of 0–300 cm soil layer was 52.2, 19.9, 11.7, 32.1, and 27.9 mm higher than that of the control R ↔ R. From 30 days to 90 days after sowing, primarily because of the rapid growth of maize, the water storage capacity of different treatments decreased significantly, and the N ↔ S treatment decreased the most. At 120 days after sowing, the water storage capacity of each treatment significantly increased, mainly because the rainfall increased and the soil moisture recovered during this period. The soil water storage was the highest in the N ↔ S treatment, which was significantly different from that of other treatments (P < 0.05). After 5 years of experiments, by analyzing the entire growth period of corn, compared with R ↔ R treatment, N ↔ S, S ↔ R, R ↔ N, N ↔ N and S ↔ S treatments average soil water storage in 0–300 cm soil layer increased by 37.8 mm, 43.5 mm, 37.8 mm, 20.8 mm and 37.6 mm, respectively.

### Soil water content during growing periods

**Figure 5:**
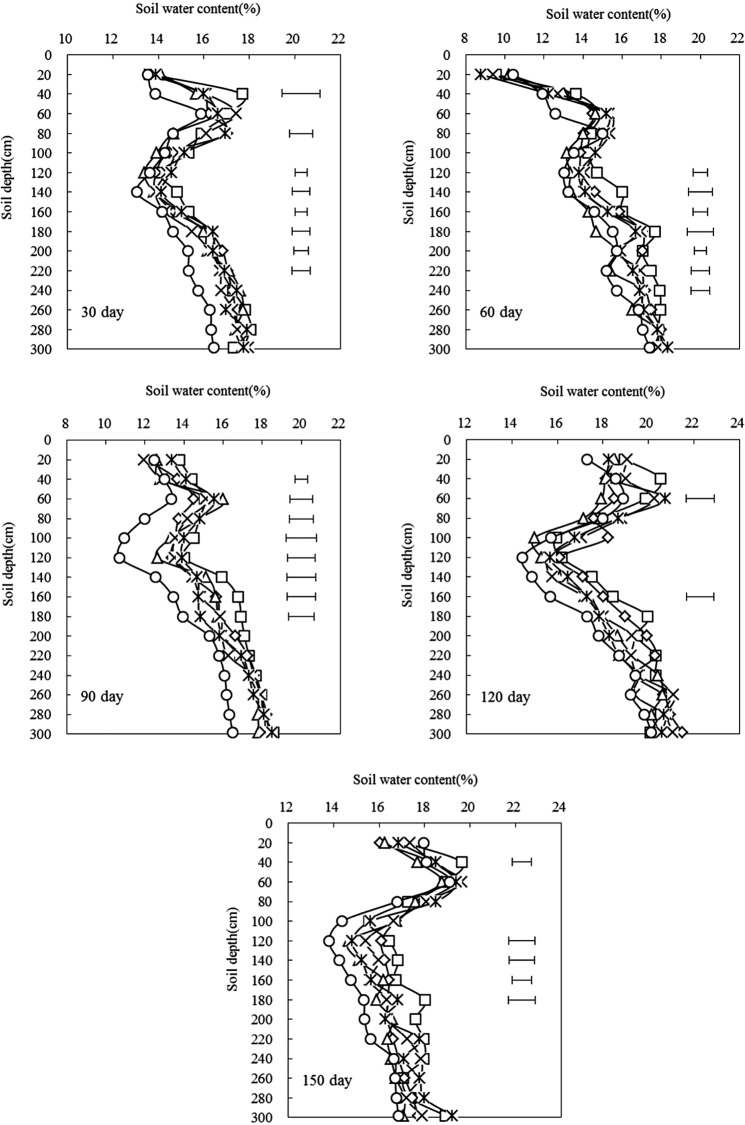
Effects of different tillage treatments on annual soil water content in 0–300 cm depth of maize field after the sowing.

Figure 5 shows the dynamic change in soil water content in the 0–300 cm soil layer under different tillage treatments during the maize growth period. The soil water content during the growth period was greatly affected by precipitation. The soil water profile characteristics of the 0–300 cm soil under each treatment were similar, that is, there was a small increase in the 0–60 cm, a decreasing trend in 60–120 cm, and an increasing trend in 120–300 cm, but both in the 40–60 cm. Soil moisture content in the soil layer was maintained at a high level, which is consistent with the trend in the soil moisture profile during rest period.

During the entire spring maize growth period, the soil water content fluctuated less during each period. Only 90 days after the maize was planted (large trumpet period), the soil water content decreased significantly, and the soil water content remained basically stable in other periods (Fig. 5). The soil moisture content fluctuated from 13.0% to 18.1% 30 days after sowing of spring maize; there were significant differences among the tillage treatments, primarily in the 20–40 cm, 60–80 cm, and 120–220 cm layers, and the treatment room. There were significant differences 60 days after sowing because of reduced rainfall at the beginning of the growing period and the need for crop growth. Compared with 30 days after sowing, the soil water content decreased, and the fluctuation range was 8.8% to 18.3%. The significant difference between the tillage treatments was mainly in the deep layer at 120–240 cm. At 90 days after sowing, the soil water content fluctuated from 10.7% to 18.5%. The significant difference between the tillage treatments was only at 20–40 cm and 40–60 cm in the soil surface, and 80–180 cm in the deep soil. After 120 days of sowing, the maize grew faster. The soil moisture in the 40–60 cm surface layer and the 140–160 cm deep soil layer was significant. During this period, the rainfall was heavy, the soil moisture was restored and the water content fluctuation range was 14.5–21.1%. At 150 days after sowing, the crop is near harvest, the growth is basically stagnant, the soil moisture content was lower than at 120 days after sowing, and the fluctuation range was 13.8–19.2%. The soil moisture content in the tillage treatment was significantly different, mainly in the soil surface layer at 20–40 cm and deep layer 120–180 cm. During the whole spring maize growth period, the soil water content under each treatment was higher than that of the control R ↔ R, and the N ↔ S treatment had the highest soil water content.

### SOC storage

**Figure 6:**
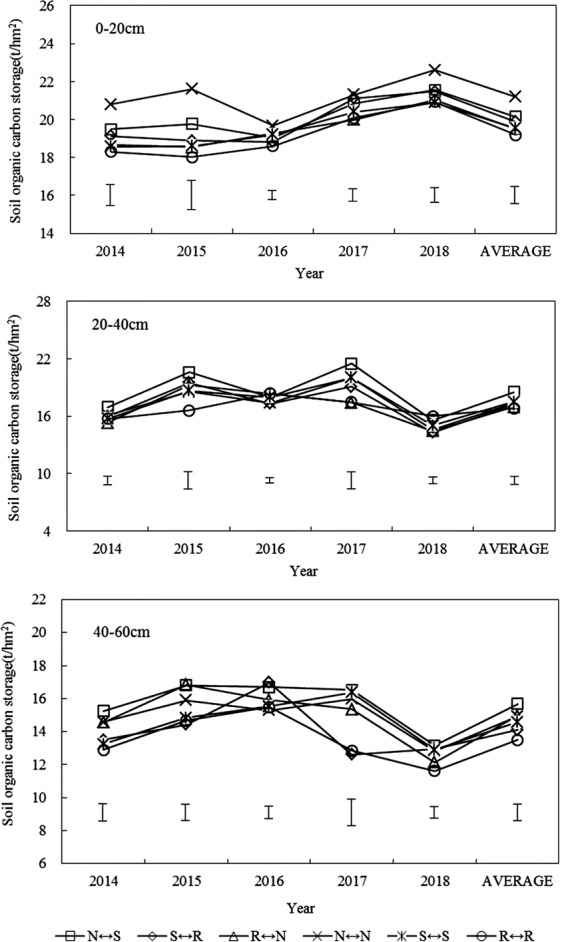
Effect of different rotational tillage treatments on soil organic carbon storage at 0–60 cm soil layer of spring corn field after harvesting.

Figure 6 shows the dynamic changes of SOC storage in 0–60 cm soil layers in different test years, with different annual differences. The SOC storage decreased with the increase of soil thickness. With the increase in experimental years, the organic carbon storage increased slightly. The SOC storage under different tillage treatments was higher than that of the control. In the 0–20 cm soil layer, the difference between the treatments was significant in the different test years. The experimental data of 5a showed that under the N ↔ N tillage mode, the average organic carbon storage in the 0–20 cm soil layer was significantly different from other treatments (P < 0.05), compared with N ↔ S, S ↔ R, R ↔ N, S ↔ S. R ↔ R increased by 5.3%, 6.6%, 8.7%, 8.5% and 10.5%, respectively. This was mainly because the N ↔ N rotation mode reduced the disturbance to the soil, and the effect of surface debris reduced the loss of organic carbon in the topsoil and mineralization, thereby increasing the SOC storage of the soil. In the 20–40 cm soil layer, the difference between the treatments was significant. The average organic carbon storage in the soil decreased by 8.1%–18.2% compared with the 0–20 cm soil layer. The SOC storage of the N ↔ S treatment was higher than that of the other treatments, with an increase range of 5.8%–10.1%, and the difference was significant. In the 40–60 cm soil layer, the difference between treatments was significant. Different tillage treatments in different trials increased the SOC storage by 4.4%–16.2%, and the organic reserves were the highest in the N ↔ S treatment. The 5a experimental study showed that the average storage of total organic carbon in the 0–60 cm soil layer was the highest, which was 54.3×10^3^ kg/ha, which means that the model has an advantage in increasing SOC accumulation in dryland.

### Crop height

**Figure 7:**
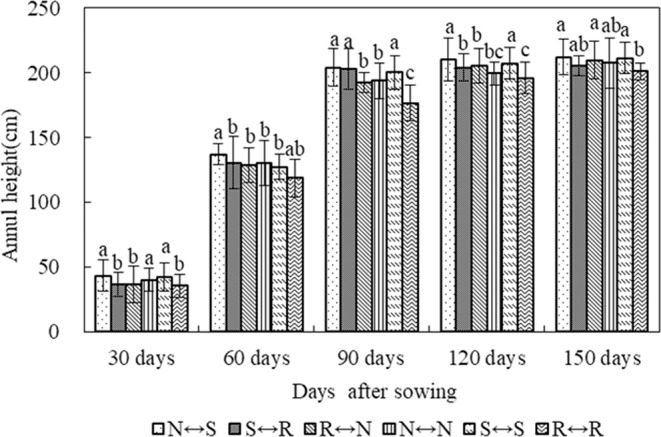
Changes in height yield of maize under different tillage treatments after the sowing of maize field in in four maize growing seasons.

In the five trial years, the average plant height variation of different treatments was different for different days after sowing (Fig. 7). In the early stage of maize growth (30–60 days), the growth rate of maize plant height was faster, the growth rate in the middle stage of fertility (60–90 days) was second, and the growth rate was slower in the late growth stage (90–150 days), and the plant height area was stable. In the early stage of fertility (30 days), the plant height of N ↔ S, N ↔ N, and S ↔ S was higher than that of S ↔ R, R ↔ N, and R ↔ R, and the difference between treatments was significant. At 60 days, the maize was treated under each treatment. The growth rate was the fastest, and the increase of plant height was 83.4–94.3 cm. The plant height of N ↔ S treated maize was the highest, which was significantly different from that of the other treatments. In the mid-fertility period (90 days), the plant height of each treated maize increased by 57.9–72.9 cm, and the plant height of each treated maize was significantly higher than that of the control R ↔ R. In the late growth stage (120 days, 150 days), the greatest plant heights of maize treated with N ↔ S and S ↔ S were 210.5 cm, 207.5 cm, 212.2 cm, and 211.2 cm, respectively. Compared with R ↔ R treatment, the increase of plant height in spring maize ranged from 2.1% to 5.4%. In the five experimental years, the differences in plant height between different growth stages of spring maize under different treatments were analyzed. N ↔ S treatment was beneficial to spring maize growth, followed by the S ↔ S treatment.

### Crop biomass

**Figure 8:**
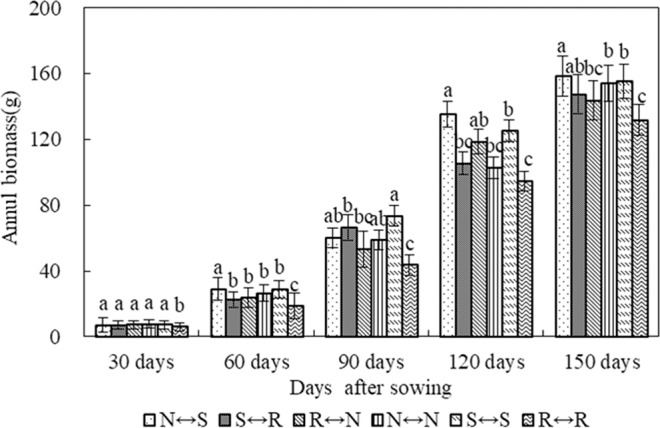
Changes in biomass yield of maize under different tillage treatments after the sowing of maize.

The inter-annual variation trend of spring maize dry matter under different tillage methods was basically consistent with its plant height. With the advancement of the maize growth period, the biomass of plants gradually increased, and the accumulation of dry matter was the largest in the middle to late growth period. The speed was gradually reduced and reached its maximum at the upcoming harvest. In the early stage of growth (30 days), the biomass per plant was significantly different from that of the control R ↔ R. In the pre-flowering period (60 days), the biomass of each treated maize treatment increased by 12.6–22.1 g compared with the initial growth stage, and the order of magnitude was N ↔ S > S ↔ S > N ↔ N > R ↔ N > S ↔ R > R ↔ R (Fig. 8). N ↔ S was significantly different from each other treatment. In the late growth stage (120 days), the increase in biomass in each treatment reached the maximum, which was 39.1 g–75.2 g compared with the middle growth stage. The increase of the treated biomass compared with the control, R ↔ R, was 8.7%–43.2% (N ↔ S). The highest treatment, followed by S ↔ S, was consistent with the trend in plant height performance. At the end of the fertility period (150 days), N ↔ S treated maize had the highest biomass of 158.5 g. The treated maize biomass was 12.1 g to 26.8 g more than that of the control, R ↔ R. The treatment of N ↔ S significantly increased the dry matter of plants.

### Crop yield and WUE

The tillage pattern will affect the physical and chemical properties of the soil, and the ultimate effect is the crop yield increase effect, which is mainly manifested in crop grain yield and WUE. In the five-year test, the interannual yield changes of spring maize with different tillage treatments and the differences between treatments are shown in Table 1. In different years, the yield of maize under different tillage treatments was higher than that of the control, R ↔ R, and the average grain yield increased by 2.1%–15.4%; the difference was significant. The difference in inter-annual production was mainly affected by rainfall during the growth period of maize. The rainfall during the growth period in 2017 was low, and the annual output was lower than that of other years. In different years, the average grain yield of different tillage treatments was N ↔ S > S ↔ S > S ↔ R > N ↔ N > R ↔ N > R ↔ R. The increased rate of R ↔ R in different years was 12.6%–23.2%.

**Table 1:** Effects of different rotational tillage systems on grain yield in 2014–2018. N ↔ S, no tillage/subsoiling; S ↔ R, subsoiling/rotary tillage; R ↔ N, rotary tillage/no tillage; N ↔ N, continuous no tillage; S ↔ S, continuous subsoiling; R ↔ R, continuous rotary tillage.

Index	Treatment	Experments years/(kg/ha)					Average
		2014	2015	2016	2017	2018	
Grain yield	N ↔ S	10310.5a	8900.8a	9253.8a	8197.9a	10038.4a	9340.2a
	S ↔ R	9509.5b	8546.6b	8925.8b	8004.9a	9297.2b	8856.7b
	R ↔ N	8820.5c	7979.3c	8277.3ab	7861.6b	8361.5c	8260.1bc
	N ↔ N	9182.6ab	8503.3b	8814.2b	7933.3a	9072.6b	8700.9ab
	S ↔ S	9602.5b	8494.9b	9153.5a	8110.2a	9273.5b	8926.8b
	R ↔ R	8386.5c	7902.9c	8218.9ab	7808.2b	8147.5c	8092.8c

Different small letters indicate significant differences (P < 0.05) within the same column.

By calculating the WUE, the utilization of soil moisture by crops under different tillage treatments could be better evaluated. In the five-year test, the inter-annual WUE changes in spring maize under different tillage treatments and the differences among treatments are shown in Table 2. The WUE performance trend in different tillage treatments was consistent with that of yield. The soil WUE of each tillage treatment was higher than that of the control, R ↔ R, with an increased range of 7.6%–41.9%. The difference between the control R ↔ R and other treatments was significant. In different years, the average WUE of different tillage treatments was N ↔ S > S ↔ S > S ↔ R > N ↔ N > R ↔ N > R ↔ R. The N ↔ S, S ↔ S, and S ↔ R tillage modes effectively improved soil WUE, and the increase rates of R ↔ R in different years were 24.6%–51.6%, 16.1%–30.5% and 14.1%–24.4%, respectively.

**Table 2:** Effects of different rotational tillage systems on WUE in 2014–2018.

Index	Treatment	Experments years/(kg/(ha/mm))					Average
		2014	2015	2016	2017	2018	
WUE	N ↔ S	23.5a	21.7a	22.8a	22.5a	23.8a	22.9a
	S ↔ R	18.9ab	19.9b	19.4b	19.2b	19.8b	19.7b
	R ↔ N	16.5bc	19.3ab	16.9bc	16.7ab	17.4bc	17.3ab
	N ↔ N	18.3ab	19.8ab	18.9ab	18.8ab	19.3b	18.9b
	S ↔ S	19.7b	20.3b	20.3b	19.5b	20.9ab	19.9b
	R ↔ R	15.5c	17.4c	15.6c	15.9c	16.2c	16.1c

N ↔ S, no tillage/subsoiling; S ↔ R, subsoiling/rotary tillage; R ↔ N, rotary tillage/no tillage; N ↔ N, continuous no tillage; S ↔ S, continuous subsoiling; R ↔ R, continuous rotary tillage. Different small letters indicate significant differences (P < 0.05) within the same column.

## Discussion

### Effect of tillage rotation on soil bulk density

The conversion of farming practices plays a key role in soil surface changes^17^. The soil bulk density at the 0–60 cm soil layer under different RT patterns showed the same trend, which was consistent with the results of Wang *et al*.^18^. Basic soil tillage measures such as NT, ST, and RT significantly improved the soil layer structure^19–21^. Rotary tillage can completely reverse the upper and lower layers of soil and bury the surface straw and residue to increase soil permeability, but the mechanical crushing must be high^22^. Subsoiling can effectively break the soil plow bottom layer, improving soil infiltration rate and strengthening soil water storage capacity^23–25^. The results of this experiment showed that the round tillage mode of NS had a better effect on soil bulk density in the 0–60 cm soil layer than did the other treatments, which was mainly because of loosening of the soil, relieving soil compacts caused by continuous NT and breaking the plow bottom formed by continuous RT. No tillage can avoid mechanical rolling of the soil, promote the formation of large aggregates in the soil, improve the stability of the aggregates, significantly improve the soil structure of the cultivated layer, and facilitate soil storage. Two cultivation measures is carried out at intervals, which not only loosens soil but also reduces the number of times the soil is crushed. This indicates that tillage effect is a long-term process, and its improvement effects on soil structure has a certain hysteresis. Long-term N ↔ S is advantageous over single NS, effectively improving the soil structure of the plow layer and significantly reducing the soil bulk density^26^. Therefore, NT and deep-slowing annual RT measures are the most effective in reducing soil bulk density and improving the soil structure, which is consistent with the results of Qin *et al*.^27^. The NT treatment over the years did not disturb the soil, but the soil was crushed in the sowing and harvesting of the crops, such that the soil bulk density increased. Soil was crushed come from the mechanized operation during the sowing and harvesting periods, and NT treatment after harvesting also increased the soil crust effect^28^. The continuous RT compacted the soil the most, had the maximum crushing strength, and the depth of RT was less than that of subsoiling, but soil bulk density did not improve.

### Effect of different tillage treatments on soil water storage and content during fallow periods

There are many studies on soil water use under protective tillage conditions, and it has become a consensus that conservation tillage is beneficial to soil water use^29^. Conservation tillage technology can reduce the soil bulk density and promote the redistribution of soil pore space in different soil layers, forming a better soil structure, thereby improving soil physical properties and soil aeration, improving rainfall infiltration and soil water storage capacity^30^. The results of this study show that compared with the traditional R ↔ R treatment, N ↔ S and N ↔ N rotation can effectively improve the soil water storage capacity during the fallow period. The improved water storage effect is the result of NT and surface straw cover. During the fallow period, the straw cover reduces the evaporation from the surface, improves rainwater storage during the fallow period, effectively preserves soil moisture^31^. No tillage and subsoiling RT improve the physical structure of the soil and enhance the water storage capacity of the soil while loosening the soil. At the same time, NS with straw cover during the fallow period can effectively reduce soil evaporation. This is consistent with results of previous studies^32^. However, the traditional RT surface is bare, the evaporation is high, and the water storage effect is poor^33,34^. With NT and RT treatments, after NT in the previous year, soil bulk density is large, and rainfall infiltration is difficult, and the subsequent RT and loose soil structure exacerbates these conditions during the fallow period. Soil water evaporation during fallow period leads to poor soil water storage effect, which is different from the result of Zhang *et al*.^35^ who studied wheat/maize rotation, this may be because of a combination of different rotation systems and fallow periods. In different years, the soil moisture content of the 0–300 cm soil layer under different tillage treatments is higher than that of traditional tillage, which is mainly because of the better effect of RT on improving soil structure, controlling weeds and pests, and increasing available nutrients. However, because of the reversal of the plow layer, the surface soil is exposed, accelerating the evaporation of soil moisture, affecting the infiltration of precipitation, and causing the soil to form a deep plow layer, which is not conducive to soil water infiltration and poor soil water storage.

### Effect of different tillage treatments on soil water storage and content during growing periods

Good soil moisture conditions are prerequisites for ensuring the germination and health of all seeds planted and high yields. In this study, at the end of the spring maize fallow period (before planting), the good water storage capacity of N ↔ S and N ↔ N during the fallow period provided better soil moisture conditions for the sowing and production of spring maize. In the early growth stage of spring maize, the rainfall is lower, and the main source of water needed for growth is from the deep layers of the soil. In the later stage of growth, the rainfall is greater, and the utilization of soil moisture by crops is reduced. This study showed that N ↔ S and N ↔ N tillage showed good water retention in the 0–300 cm soil layer during the entire growth period of maize, whereas the water conservation was relatively poor for R ↔ R tillage. The water retention effect is mainly attributed to straw mulching, which can effectively reduce the evaporation of soil moisture between crops, increase water storage, and reduce the water deficit. In the spring maize production season, the soil moisture storage and content under other treatments were higher than those of the control treatment, which was mainly because the maize growing season coincided with the summer season. In the R ↔ R treatment, the soil surface was loose, there was no straw cover, resulting in high soil evaporation. The performance was consistent with the fallow period. The soil moisture effect was better under NT and deep loosening combined with straw mulching. The main advantage of NT is straw mulching, which can improve the upper soil structure, promote underwater seepage, reduce evaporation of water and increase the moisture content of the upper layer of soil^36,37^. The key to ST tillage is to form a “virtual and real coexistence” vertical farming layer, the “virtual” part of the plow bottom is broken, which is conducive to fully storing rainwater and increasing the deep water storage, the “real” part is good for water retention, water lifting, and water supply.

### Effect of different tillage treatments on SOC content

Soil carbon sequestration is mainly achieved by reducing the soil carbon pool decomposition and increasing soil carbon pool input. Long-term no-tillage can reduce the disturbance to the soil, reduce the soil organic carbon mineralization rate, and thus maintain the soil organic carbon storage, which is consistent with the results of Lal *et al*.^38^ who studied the11 years of positioning experiments showed that the soil organic carbon storage in the long-term no-tillage mode is higher than that in other rotation modes, showing an enrichment phenomenon in the 0–20 cm soil layer. In addition, the long-term NT mode can increase the number of upper roots of spring maize, thereby increasing soil carbon. Qin *et al*.^39^ pointed out that no-tillage increased the root length density of 0–5 cm summer corn compared with traditional rotary tillage, which increased the input of carbon pool. The deep-soil treatment mechanically cultivated the soil, so that the soil of the tillage layer was evenly mixed, and the distribution characteristics of the residues and other substances were changed, thereby increasing the organic carbon storage of the lower soil layer, which is consistent with the results of Zhuang *et al*.^40^. The long-term single farming mode, such as traditional single rotation, results in frequent mechanical disturbances to the soil, destroying the agglomerate structure of the soil layer, resulting in increased soil activity and increasing the mineralization rate of soil carbon. In this study, the equivalent depth method is used to calculate SOC storage. Consistent with the results of most previous studies, NT can significantly increase the surface organic carbon storage, but Hou *et al*.^41^ showed that long-term NT did not significantly change the characteristics of cultivated black soil, which may be related to the texture of the local soil sample. This experimental study showed that the soil organic carbon storage in each soil layer increased significantly under the six tillage modes (P < 0.05). The integrated tillage modes, such as R ↔ S and S ↔ S are mainly due to farming measures disturbing the soil of the plow layer, increasing soil aeration and contact between the soil and the residue, thereby accelerating the conversion of organic carbon. The conclusion consistent with the results of Joseph and Kristian^42^ who believes that NT and subsoiling integrated rotation mode are superior to traditional tillage and can increase soil carbon sequestration capacity, mainly because NT can increase the organic carbon storage of surface soil, subsoiling changes the distribution characteristics of SOC in the plow layer, increases the organic carbon storage of the corresponding tillage depth, and evenly distributes organic carbon, all of which can be beneficial to the formation of plow layer structure and crop growth.

### Effect of different tillage treatments on crop biomass and height

Different farming methods have different effects on crop growth and development. For example, straw mulching can effectively increase soil moisture storage and have a positive effect on crop production and development. The NT farming mode can effectively increase soil moisture and reduce unnecessary waste. Subsoiling promotes a good retention of soil moisture to encourage the elongation of crop roots and effectively absorb water and fertilizer, which is conducive to increasing dry matter accumulation and increasing crop yield. Through the implementation of conservation tillage in different regions, many scholars have found that the round tillage model can effectively improve the dry matter accumulation of crops compared with traditional tillage. For example, Hou *et al*.^41^ implemented an effective combination of subsoiling and NT in the agricultural area of Guyuan, Ningxia, which significantly increased the biomass of crop wheat, which is consistent with the results of this study. This study showed that the average plant height and biomass of spring maize in the five trials in 2014–2018 showed that the plant height and biomass of spring maize under N ↔ S treatment increased significantly compared with that of other treatments, followed by the S ↔ S treatment. This is mainly because the combination of NT and subsoiling can effectively reduce water evaporation, reduce soil compaction and disturbance, improve soil structure, build a good soil environment, and provide suitable seed beds for seed germination and growth.

### Effect of different tillage treatments on crop yield and WUE

Soil production performance is a comprehensive reflection of soil water, fertilizer, gas, heat coordinated supply and crop economic yield. The same farming mode will vary in different years due to different farming methods adopted, and its crop yield and water use efficiency (WUE) will be different.The purpose of dryland soil cultivation is to establish soil environmental conditions suitable for crop growth, enhance water storage and preservation capacity, and promote crop yield increase. If a reasonable farming mode is adopted, the soil quality can be improved, soil bulk density and compaction degree can be reduced, and a suitable seed bed for maize growth and development can be provided, which is beneficial for the germination and growth of maize kernels, thereby increasing the yield. The tillage mode uses different farming methods in different years. Its single-year crop yield and WUE not only evaluate the advantages and disadvantages of the round farming mode but also comprehensively compare the model cycles. Wang *et al*.^18^ showed that the subsoiling-rotary tillage rotation mode is the best for increasing the yield and income of winter wheat–spring maize rotation system in the Loess Plateau, followed by the NT–subsoiling, which may be mainly related to the crop planting system. This study showed that the yield of corn treated with R ↔ R was the lowest under different farming modes in different years, and the yield of grain and WUE benefited from the crop treated with N ↔ S. The system could break the plow pan and promote precipitation, seepage, reduce soil bulk density, increase SOC content, uniformly distribute nutrients in the soil tillage layer, improve water storage, and increase moisture retention and fertility. The N ↔ S was followed by S ↔ S, which was consistent with the results of Joseph and Kristian^42^.

## Materials and Methods

### Site description

**Figure 9:**
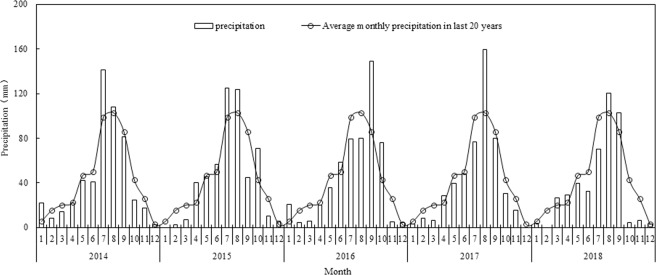
Precipitation during 2014–2018.

The test was conducted at Fuping, located in Yucun Village, Ducun Town, Fuping County, Weinan City, Shaanxi Province (109 °11′N, 34 °42′E). The area is a warm temperate semi-arid climate zone with an average annual rainfall of 472.97 mm. The annual amount and variability of rainfall was large. Rainfall from July to September accounts for 49% of the annual rainfall, and the annual evaporation is 1000–1300 mm. The annual average temperature is 13.4 °C, and the climatic conditions can meet the needs of crop growth, which is consistent with the main growth period of spring maize. Precipitation over numerous years is shown in Fig. 9. The soil quality of the test site is good, and the soil layer thickness is better. The soil type is the dark loessial soils that is common in this area. The planting system for this experiment was one year of spring maize, and the soil physical and chemical properties before the test are shown in Table 3.

**Table 3:** The soil basic chemical properties of the pretreatment.

Soil layer (cm)	Soil organic matter (g/kg)	Total N content (g/kg)	Total P content (g/kg)	Total K content (g/kg)	Available P content (mg/kg)	Available K content (mg/kg)
0–20	4.34	1.22	0.55	5.95	3.47	145.3
20–40	1.58	0.85	0.19	5.49	2.12	136.2
40–60	1.29	0.81	0.07	5.35	1.79	131.1

### Field experiment design

A five-year (2013–2018) spring maize continuous cropping experiment was conducted. Rotary tillage without corn stalk addition was carried out before the start of the experiment, and the rotary tillage depth was 15–20 cm.,this process was called BT. Under the conditions of spring maize (Jincheng 508, Variety source: Jin 522 × Jin 865) cultivation, the straw was pulverized and returned to the field immediately after harvest. The three different soil cultivation measures implemented were as follows: (1) no tillage treatment (NT)-after the maize was harvested, the straw was pulverized and added to the soil to cover the surface, without any soil cultivation measures; this formed the straw surface cover during the winter fallow period. (2) subsoiling treatment (ST)-after the maize was harvested, the straw was pulverized and added to the soil to cover the surface. A single shovel was 30–35 cm deep and 40–60 cm wide; the pulverized straw formed the surface cover during the winter fallow period. (3) rotary tillage treatment (RT)-immediately after the maize was harvested, the straw was pulverized and added to the soil to cover the surface. A single plow was used to turn 15–20 cm, to bury the straw, exposing the soil surface during the winter fallow period. Other than spraying herbicide once during the fallow period, the soil was not disturbed before sowing. In this study, the six tillage treatments were composed of three cultivation measures: NT/ST rotation (N ↔ S, no tillage was applied in first year and rotated with subsoiling in second year), ST/RT rotation (S ↔ R, subsoiling was applied in first year and rotated with rotary tillage in second year), RT/NT (R ↔ N, rotary tillage was applied in first year and rotated with no tillage in second year), NT/NT rotation(N ↔ N, no tillage was applied in first year and rotated with no tillage in second year), ST/ST rotation(S ↔ S, subsoiling was applied in first year and rotated with subsoiling in second year) and RT/RT rotation(R ↔ R, rotary tillage was applied in first year and rotated with rotary tillage in second year). The experimental area was a randomized block design with three replications. Each plot was 5 m wide and 12 m long. The specific soil cultivation during test period is shown in Table 4.

**Table 4:** Sequence of soil rotational tillage systems from 2014 to 2018.

Rotational system	Before treatment	Year				
		2014	2015	2016	2017	2018
N ↔ S	BT	NT	ST	NT	ST	NT
S ↔ R	BT	ST	RT	ST	RT	ST
R ↔ N	BT	RT	NT	RT	NT	RT
N ↔ N	BT	NT	NT	NT	NT	NT
S ↔ S	BT	ST	ST	ST	ST	ST
R ↔ R	BT	RT	RT	RT	RT	RT

N ↔ S, no tillage/subsoiling; S ↔ R, subsoiling/rotary tillage; R ↔ N, rotary tillage/no tillage; N ↔ N, continuous no tillage; S ↔ S, continuous subsoiling; R ↔ R, continuous rotary tillage; NT, no tillage; ST, subsoiling; RT, rotary tillage; BT, rotary tillage without corn stalk addition was used before the experiment.

In this experiment, spring maize was sown in mid-March with a seeding rate of 75 kg/ha. The seeds were planted 60 cm apart, with a planting density of 67,500 plants/ha. Fertilizer was broadcast applied before sowing. The fertilization amount was N 150 kg/ha, P_2_O_5_ 120 kg/ha, and K_2_O 90 kg/ha. The nitrogen, phosphorus, and potassium fertilizers were urea, diammonium phosphate, and potassium sulfate, respectively. The specific crop cultivation situation in 2013–2018 is shown in Table 5.

**Table 5:** Planting details in winter wheat-spring maize field during 2014–2018.

Year	Crop	Plangting date	Harvest Date	Variety	Precipitation/mm
2013–2014	Fallow period	—	—	—	155.3
2014	Spring maize	2014-04-21	2014-09-18	Jincheng 508	426.4
2014–2015	Fallow period	—	—	—	55.6
2015	Spring maize	2015-04-18	2015-09-23	Jincheng 508	408.1
2015–2016	Fallow period	—	—	—	150.1
2016	Spring maize	2016-04-19	2016-09-16	Jincheng 508	404.4
2016–2017	Fallow period	—	—	—	120.1
2017	Spring maize	2017-04-23	2017-09-21	Jincheng 508	412.0
2017–2018	Fallow period	—	—	—	86.8
2018	Spring maize	2018-04-22	2018-09-24	Jincheng 508	386.7

### Measurements

#### Soil bulk density

After the spring maize was harvested, a standard ring cutter (5 cm in height and 5.04 cm in diameter) was used for soil sampling. The depth of the soil collected in each plot was 0–60 cm, which was the main depth of soil cultivation and fertilization treatment. The sampling interval was 0–20, 20–40, and 40–60 cm to reveal the difference in tillage and crop root growth on the soil bulk density of different soil layers. According to the shape of the test plot, five sampling points were selected using the diagonal method, and undisturbed soil samples from three soil layers were collected, sealed, and returned to the laboratory. The soil samples were dried (105 °C, 24 h) to determine the soil bulk density.

1 $${\rm{BD}}({\rm{g}}/{{\rm{cm}}}^{3})=({\rm{WS}}-{\rm{W}})/{\rm{V}}$$

where BD is soil bulk density, g/cm^3^; WS is cutting ring and soil dry mass, g; W is cutting ring knife mass, g; and V is ring knife volume, cm^3^.

#### Soil water

The soil moisture of the 0–300 cm soil layer was measured by the drilling and drying method at 30 days, 60 days, 90 days, 120 days, and 150 days and during the winter fallow period after sowing of the spring maize. The sampling interval was 20 cm, and each plot was used. The soil water content was measured at the beginning of october and the beginning of april in the fallow period. Calculation of soil water storage and water use efficiency are as follows:

2 $${\rm{SW}}=({{\rm{M}}}_{1}-{{\rm{M}}}_{2})/{{\rm{M}}}_{2}\times 100 \% $$

where SW is the soil moisture content; %; M_1_ is the wet soil weight, g; and M_2_ is the dry soil weight, g.

3 $${\rm{W}}={{\rm{SW}}}_{{\rm{i}}}\times {{\rm{P}}}_{{\rm{i}}}\times {{\rm{H}}}_{{\rm{i}}}\times 10/100$$

where W is the soil water storage capacity, mm; SW_i_ is the i-th layer soil mass water content, %; Pi is the i-th layer soil volume mass, g/cm^3^; and Hi is the i-th layer soil thickness, cm.

4 $${\rm{ET}}={{\rm{R}}}_{2}+\Delta W$$

where ET is the crop water consumption, mm; R^2^ is the crop growth period precipitation, mm; and ΔW is the change in soil water storage during the calculation period, mm.

5 $${{\rm{WUE}}}_{1}={\rm{GY}}/{\rm{ET}}$$

where WUE_1_ is the grain use water use efficiency, kg/(ha·mm); GY is the maize grain yield, kg/ha; and ET is the crop water consumption, mm.

#### SOC storage

The soil samples were collected immediately after the harvest of spring maize each year. The soil depth of each plot was 0–60 cm, and the sampling interval was 20 cm. Five sampling points were set by the diagonal method, and one sample was mixed with every five replicate samples of the same depth. The sample was taken back to the laboratory for natural air drying, after removing the gravel, plant roots, residue, and any other debris. SOC was determined using a potassium chromite (K_2_Cr_2_O_7_) oxidation exogenous heating method (GB7857–1997) after passing through a 0.25 mm sieve. For using the equal depth method, the depth of soil carbon storage was considered as 20 cm for each treatment. Based on Liang *et al*.^43^, the following formula was used:

6 $${{\rm{SOC}}}_{{\rm{storage}}{\rm{in}}{\rm{depth}}}=2\times {\rm{BD}}\times {\rm{C}}$$

where SOC_storage in depth_ is the soil carbon storage per unit area of equal depth (20 cm), kg/ha; BD is the soil bulk density, g/cm^3^; C is organic carbon content in the 0–20, 20–40, and 40–60 cm soil layer under each treatment, g/kg.

#### Crop height and biomass

After sowing and emergence of spring maize, 30 representative plants with uniform growth were selected from each plot, and the plant height was measured at 30 days, 60 days, 90 days, 120 days, and 150 days after planting. Dry weight of the crop was determined, and five plants were repeatedly sampled in each plot and dry weight was determined using a drying method at 105 °C for 1 h and drying again at 70 ° C for 72 h.

#### Yield

Three samples were randomly selected from 9 m^2^ of each plot to determine maize yield. The sample was manually threshed, and the yield was calculated after air drying.

### Statistical analysis

Differences between treatments was assessed by randomized block analysis by using two-way ANOVA. When the soil dynamics under different tillages in the growth and rest periods were compared, a one-way ANOVA followed by Duncan’s multiple range test was used. Differences were considered significant at P < 0.05. All experimental data, including soil bulk density, soil water, crop biomass, SOC, yield, and WUE were analyzed using SPSS statistical package v.20.0 (SPSS Inc., Chicago, IL), and the data in Figs. 3–9 were generated using SigmaPlot 12.5 (Systat Software, Inc.).
